# Histone deacetylases: potential therapeutic targets for idiopathic pulmonary fibrosis

**DOI:** 10.3389/fcell.2024.1426508

**Published:** 2024-08-13

**Authors:** Hai-peng Cheng, Shi-he Jiang, Jin Cai, Zi-qiang Luo, Xiao-hong Li, Dan-dan Feng

**Affiliations:** ^1^ Department of Pathology, The Second Xiangya Hospital, Central South University, Changsha, Hunan, China; ^2^ Hunan Clinical Medical Research Center for Cancer Pathogenic Genes Testing and Diagnosis, Changsha, Hunan, China; ^3^ Department of Physiology, Xiangya School of Medicine, Central South University, Changsha, Hunan, China; ^4^ Hunan Key Laboratory of Organ Fibrosis, Central South University, Changsha, Hunan, China

**Keywords:** histone deacetylase, idiopathic pulmonary fibrosis, histone acetylation, fibroblasts, HDAC inhibitors

## Abstract

Idiopathic pulmonary fibrosis (IPF) is a chronic progressive disease of unknown origin and the most common interstitial lung disease. However, therapeutic options for IPF are limited, and novel therapies are urgently needed. Histone deacetylases (HDACs) are enzymes that participate in balancing histone acetylation activity for chromatin remodeling and gene transcription regulation. Increasing evidence suggests that the HDAC family is linked to the development and progression of chronic fibrotic diseases, including IPF. This review aims to summarize available information on HDACs and related inhibitors and their potential applications in treating IPF. In the future, HDACs may serve as novel targets, which can aid in understanding the etiology of PF, and selective inhibition of single HDACs or disruption of HDAC genes may serve as a strategy for treating PF.

## 1 Introduction

Pulmonary fibrosis (PF) is a progressive lung disease with high mortality and disability rates worldwide because other effective treatments aside from lung transplantation are unavailable (Richeldi et al., 2017). Its common manifestation is progressive lung scarring and usual interstitial pneumonia, which can lead to respiratory failure and death (Martinez et al., 2017). With the continuous spread of SARS-CoV-2, the cumulative number of infections worldwide has exceeded 130 million. PF is a potential long-term complication of coronavirus infection (Cocconcelli et al., 2021; Faverio et al., 2022). Therefore, now that the COVID-19 pandemic is controlled, PF prevention and treatment are expected to become an important global issue.

PF involves a complex interplay of genetic susceptibility, aging, and environmental factors; however, its exact etiology remains unclear. Large-scale studies have demonstrated that 75% of individuals with PF have a history of smoking and that genetic background plays a critical role in PF development (Wolters et al., 2014; Wolters et al., 2018). Epigenetic factors also contribute to PF development (Coward et al., 2009; Coward et al., 2010; Sanders et al., 2012; Yang et al., 2014; Sehgal et al., 2022). Specifically, epigenetic mechanisms alter gene expression through DNA methylation, histone modifications, and non-coding RNAs. The HDAC-derived deacetylated chromatin is a crucial driving force behind the influence of epigenetics on the pathophysiology of PF (Korfei et al., 2022).

Several histone deacetylases (HDACs), including HDAC1, HDAC2, HDAC3, HDAC4, HDAC5, HDAC6, HDAC7, and HDAC8, play vital roles in PF progression (Shan et al., 2008; Pang and Zhuang, 2010; Huang et al., 2013; Khalil et al., 2015; Rubio et al., 2019; Zhang et al., 2020; Jeong et al., 2022; Hua et al., 2023). However, the relationship between newly discovered HDAC subtypes, such as HDAC9, HDAC10, and HDAC11, and PF progression has received little attention. Furthermore, pan-HDAC inhibitors have shown therapeutic potential in preclinical models of lung fibrosis (Zhang et al., 2013; Ye et al., 2014; Ota et al., 2015; Rao et al., 2016; Korfei et al., 2018). Selective effects on single HDACs, such as HDAC3, HDAC6, and HDAC8, may trigger an effective therapeutic response (Saito et al., 2017; Saito et al., 2019; Chen et al., 2021). However, the roles, mechanisms, and inhibitors of HDACs in PF remain unclear. Thus, this review aims to discuss the role of each HDAC subtype and its inhibitors in PF development. It serves as a basis for using HDACs as novel molecular targets and selective inhibition of single HDACs as a potential treatment for PF. Due to their unique structural and mechanistic properties, sirtuins stand apart from the other histone deacetylase classes, including HDAC1-11. Given that sirtuins do not rely on Zn^2+^ for their catalytic activity and are thus unaffected by the common Zn^2+^-dependent HDAC inhibitors, this review will not encompass a discussion on the sirtuin family members.

## 2 Overview of HDACs

Transcription regulation in eukaryotes occurs in the chromatin environment and is deeply influenced by posttranslational modifications of histones, such as methylation, phosphorylation, and acetylation (Attwood et al., 2002; Kim et al., 2022). The steady state of acetylation depends on the balance between histone acetyltransferases and HDACs. HDACs contain 18 genes and are classified as I–IV based on their respective yeast homologs. Classes I, II, and IV form the “classic” HDACs, which include 11 family members, namely, HDAC1–11. Class III, called sirtuins, consists of seven family members, namely, SIRT1–7. HDAC1–11 require zinc (Zn^2+^) for catalysis, whereas SIRT1–7 relies on oxidized nicotinamide adenine dinucleotide (Haberland et al., 2009; Witt et al., 2009). The Zn^2+^-dependent HDACs (Classes I, II, and IV) are recognized as “classical HDACs” and common targets for therapy. However, sirtuins are structurally and mechanistically distinct from these Zn^2+^-dependent HDAC classes and are not inhibited by the widely used Zn^2+^-dependent HDAC inhibitors. As a result, the Class III sirtuins and their inhibitors are not in the scope of this review.

**FIGURE 1:**
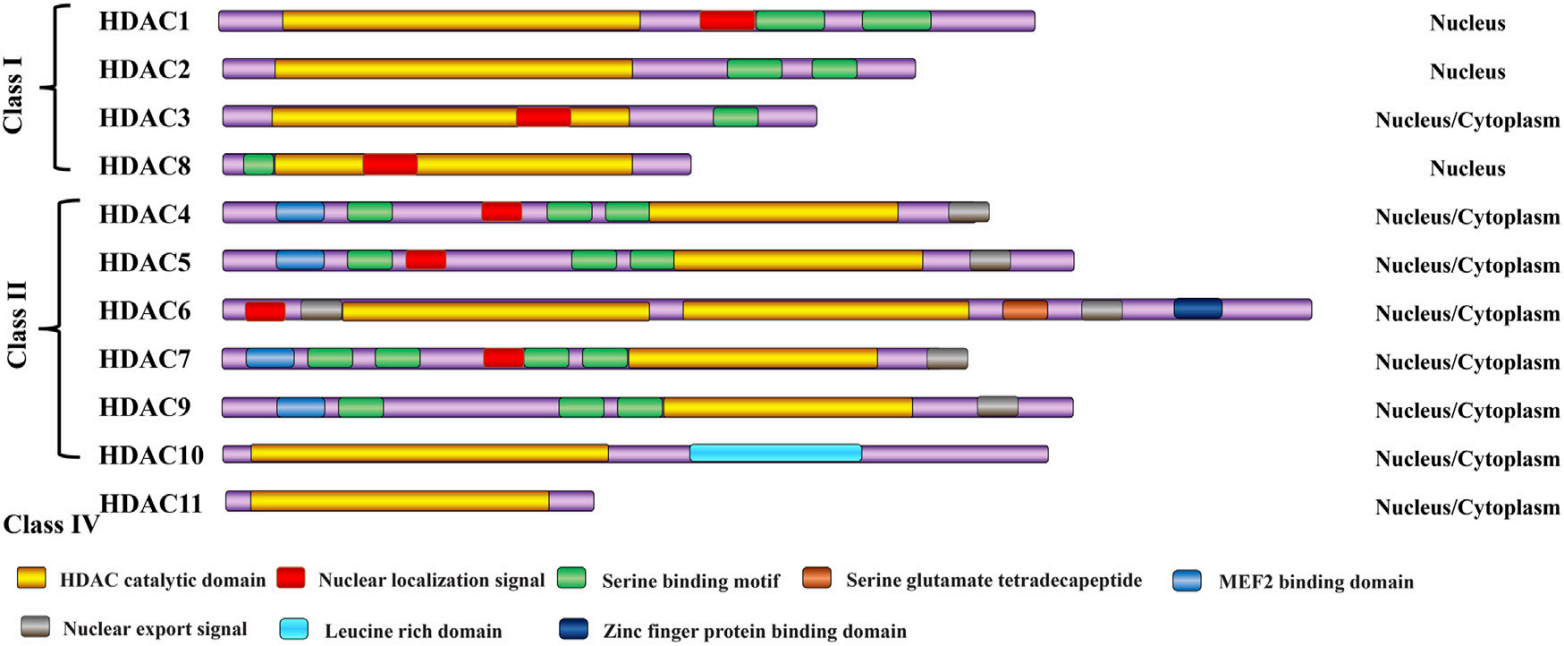
Classification of HDAC enzymes. All HDAC proteins of the histone deacetylase family share a common ancestor, resulting in similar 3D structure, function, and sequence homology. H3/H4: histone H3/H4; Ac: acetylation.

Class I HDACs, including HDAC1–3 and HDAC8, are mainly located in the nucleus and are widely expressed in various tissues. The deacetylation of nucleosomal histones by Class I HDACs is primarily carried out through the formation of enzymatically active complexes (Xu et al., 2007; Sanaei and Kavoosi, 2019). Class II HDACs, including HDAC4–7, HDAC9, and HDAC10, are located in the cytoplasm but can shuttle to the nucleus (Xu et al., 2007; Sanaei and Kavoosi, 2019). HDAC11, the sole representative of Class IV enzymes, is expressed in various tissues such as the brain, heart, kidney, testis, and skeletal muscle, where it is primarily localized in the nucleus. Notably, HDAC11 stands out for its exceptional catalytic efficiency as a fatty acid acylase, harboring a catalytic activity center that is shared by both Class I and Class II enzymes (Chen et al., 2022). HDAC proteins in the histone deacetylase family share a common ancestor, resulting in similar 3D structure, function, and sequence homology. HDAC typically has a 350-amino-acid core domain with two conserved isoforms: the N-terminal and central domains. In contrast, the C-terminal domain is more diverse. The HDAC domain contains catalytic sites, like zinc ions and arginine residues, essential for its activity (Figure 1). Moreover, HDAC can form complexes with regulatory proteins and transcription factors (Wang et al., 2016).

## 3 Roles of HDACs in PF development

### 3.1 Class I HDACs in PF

#### 3.1.1 HDAC1 and HDAC2

The human HDAC1 gene is located on chromosome 1p34, whereas the mouse HDAC1 gene is located on chromosome 4D2. The HDAC1 protein comprises 482 amino acids (de Ruijter et al., 2003). The human HDAC2 gene is located on chromosome 6q21, whereas the mouse HDAC2 gene is located on chromosome 10B1. HDAC1 and HDAC2 share 85% global sequence identity (Segré and Chiocca, 2011). Moreover, they share similar structures and many binding partners in macromolecular complexes (Brunmeir et al., 2009; Segré and Chiocca, 2011). The nuclear localization of HDAC1 and HDAC2 suggests that their primary role is regulating gene expression (Johnstone, 2002). HDAC1 and HDAC2 mediate lysine deacetylation within histone tails. This process enhances the affinity of histones for the negatively charged DNA backbone, effectively suppressing transcription by impeding the access of transcriptional machinery and transcription factors to gene promoter regions. This phenomenon has led to the classical understanding of the role of HDAC1 and HDAC2 in suppressing transcription. Furthermore, the expression levels of HDAC1 and HDAC2 are significantly elevated in fibrotic lesions of idiopathic pulmonary fibrosis (IPF) lung tissues and primary IPF fibroblasts (Korfei et al., 2015). This finding has also been demonstrated in numerous rodent models of PF (Huang et al., 2013; Li et al., 2017). Several studies have reported the antifibrotic properties of HDAC inhibitors. Primary IPF fibroblasts treated with the pan-HDAC inhibitor LBH589 and the Class I HDAC inhibitor valproic acid (VPA) can considerably reduce the profibrotic and antiapoptotic phenotypes owing to the regulatory effect of Class I HDACs, such as HDAC1, HDAC2, and/or HDAC3, on the expression of various key molecules that are antiapoptotic and profibrotic in lung fibroblasts (Korfei et al., 2015). Furthermore, LBH589 inhibits HDACs and inactivates HDAC1 and HDAC2 via significant proteolysis, which abrogates profibrotic STAT3 phosphorylation and activates downstream signaling pathways, consequently decreasing cell proliferation, survivin expression, and the expression of genes associated with extracellular matrix (ECM) in IPF fibroblasts (Korfei et al., 2018). Meanwhile, the HDAC inhibitor suberoylanilide hydroxamic acid induces the apoptosis of primary myofibroblasts, decreases bleomycin (BLM)- stimulated murine model lung fibrosis, and improves lung function by modifying the susceptibility of myofibroblasts to apoptosis (Sanders et al., 2014). The proliferation of IPF fibroblasts is effectively reduced, and the expression of interstitial collagen is suppressed *in vitro* through the selective inhibition of spiruchostatin A, which exhibits a distinct activity profile toward Class I HDACs (Davies et al., 2012). Profibrotic signaling through STAT3 phosphorylation and activation is associated with epigenetic alterations while inducing cell cycle arrest and apoptosis in IPF fibroblasts (Korfei et al., 2018), and it may be enhanced by the lysine deacetylation of STAT3 depending on HDAC1, HDAC2, and HDAC3 (O'Shea et al., 2005; Gupta et al., 2012). Consistently, BG45 is a novel Class I HDAC inhibitor that participates in the activation of the JAK2/STAT3 pathway (Tang et al., 2018). Taken together, these studies indicate that the upregulation of HDAC1 and HDAC2 expression is linked to survivin expression, cell proliferation, myofibroblast transformation, and ECM-associated genes in IPF fibroblasts.

#### 3.1.2 HDAC3

The human HDAC3 gene is located on chromosome 5q31, whereas the mouse HDAC3 gene is located on chromosome 18B3 (de Ruijter et al., 2003). The predicted amino acid sequence of HDAC3 comprises a 49 kDa molecular mass open reading frame, encompassing 428 amino acids (Yang et al., 2002). However, information about the role of HDAC3 in IPF is limited. Korfei et al. found that HDAC3 expression is significantly upregulated in alpha-smooth muscle actin (α-SMA) positive-expressing myofibroblasts and ciliated bronchial cells in IPF (Korfei et al., 2015). Recent studies have highlighted that microenvironmental signals, particularly stiffness, in progressive fibrosing interstitial lung diseases serve as media to integrate the contribution of epigenetics to direct persistent fibroblast activation (Toscano-Marquez et al., 2023). Matrix stiffness reduces nuclear HDAC3 expression in fibroblasts, resulting in chromatin opening and hyperacetylation. This phenomenon consequently upregulates the expression of profibrotic genes, including *Col1A1*, *α-SMA*, and *p21* (Toscano-Marquez et al., 2023). Additionally, HDAC3 promotes epithelial–mesenchymal transition (EMT), inflammation, and PF development by activating Notch intracellular domain one and signal transducer and activator of transcription one signaling to upregulate the expression of inflammasome components, including AIM2 and ASC (Zheng et al., 2022). Aberrant HDAC3 elevation and its suppression of Nrf2 decrease the expression of catalase and superoxide dismutase 3, leading to an insufficient capacity of antioxidant stress and antifibrosis capacities and consequently promoting PF progression (Chen et al., 2021). HDAC3 regulates the hypoxia-induced EMT in response to the increasing of hypoxia-inducible factor-1α via the AKT signaling pathway and HDAC3-miR224-FOXA1 axis, enhancing the migratory and invasive properties of fibroblasts (Jeong et al., 2022). HDAC3 upregulates the expression of miR-19a-3p-mediated interleukin 17 receptor A. This phenomenon downregulates the expression of fibrosis marker genes, such as COL1A1, COL3A1, and FN, which facilitate the development of rheumatoid arthritis-associated interstitial lung disease or fibrosis (Yuan et al., 2020). Recent studies have shown that increased expression of histone deacetylase three in alveolar type 2 epithelial cells promotes EMT and PF progression. This process is associated with the activation of the TGF-β1/SMAD3 signaling pathway (Xiong et al., 2023). Taken together, these findings indicate that the abnormal overexpression of HDAC3 in fibroblasts/myofibroblasts plays a crucial role in mediating fibrogenic signaling, particularly in IPF, and can be mitigated by the targeted inhibition of HDAC3.

#### 3.1.3 HDAC8

The human HDAC8 gene is located on chromosome Xq13, whereas the mouse HDAC8 gene exists on chromosome XD (de Ruijter et al., 2003). HDAC8 consists of 377 amino acids (Buggy et al., 2000). The expression of HDAC8 increases in IPF lung cell types, such as fibroblast foci, smooth muscle cells, and vascular smooth muscle cells (Korfei et al., 2015). Furthermore, HDAC8 is expressed in the cytoplasm and nucleus of the aforementioned cell types (Korfei et al., 2015). However, little is known about the role of HDAC8 in PF. Previous studies found that HDAC8 most efficiently influences the formation of the SMA cytoskeleton and the contractility of smooth muscle cells (Waltregny et al., 2004; Waltregny et al., 2005). Zhao et al. have recently reported that treatment with the HDAC8 selective inhibitor PCI34051 and knockdown of HDAC8 expression mitigate cardiac fibrosis by inhibiting the tumor growth factor (TGF)-β1/Smad2/3 pathway in rat cardiac fibroblasts (Zhao et al., 2022). Notably, silencing HDAC8 can significantly inhibit the activation of the TGF-β signaling pathway and suppress ACTA2 expression in human skin fibroblasts (Glenisson et al., 2007). Moreover, the selective inhibition of HDAC8 has recently been verified as an effective therapeutic schedule in BLM-induced PF (Saito et al., 2019). Shigeki et al. reported that the specific deacetylation of structural maintenance of chromosomes protein three by HDAC8 induces the inhibition of antifibrotic PPARγ-signaling, production of ECM proteins, and formation of stress fiber in TGF-β-stimulated IPF fibroblasts. This process can be abrogated by silencing HDAC8 with RNAi or using the HDAC8 selective inhibitor NCC170 (Saito et al., 2019). These studies suggest a need for further research on HDAC8 as a risk factor for fibrotic lung disease. A novel two-stage screen platform has recently been reported to accelerate the development of HDAC6, HDAC8, or dual HDAC6/8 inhibitors, which can alleviate PF progression in various animal models (Yu et al., 2023).

In conclusion, these studies consistently demonstrate the potent antifibrotic effect of Class I selective HDAC inhibitors in PF. This evidence further highlights the key role of Class I HDACs in mediating profibrotic signal transduction in progressive fibrosing interstitial lung diseases.

### 3.2 Class II HDACs in PF

#### 3.2.1 HDAC4

The human HDAC4 gene, which comprises 1084 amino acids, is located on chromosome 2q37, whereas the mouse HDAC4 gene exists on chromosome 1D1 (Wang et al., 2014). The localization of HDAC4 is shuttling between cytoplasm and nucleus based on the state of phosphorylation; for instance, dephosphorylated HDAC4 is translocated to the nucleus (Wu et al., 2016).

Aberrant expression of HDAC4 drives fibrogenesis via the HDAC4-miR-206-MRTF-A axis in hepatic stellate cells (Han et al., 2017). Furthermore, the pan-HDAC inhibitor trichostatin A inhibits TGF-β1-mediated fibroblast-myofibroblast transdifferentiation, and this process is dependent on the cytoplasmic localization of HDAC4 and requires the phosphorylation of Akt but not SMAD2/3 (Guo et al., 2009). Silencing HDAC4 efficiently abrogates the TGF-β-induced differentiation of fibroblasts into myofibroblasts by enhancing endogenous repressors of the TGF-β signaling pathway instead of Smad7 (Glenisson et al., 2007). Additionally, TGF-β increases NADPH oxidase 4-derived reactive oxygen species production and promotes HDAC4 nucleus-to-cytoplasm translocation, exerting profibrotic effects by promoting fiber formation and enhancing cell contraction in lung fibroblasts (Guo et al., 2017). Moreover, silencing HDAC4 upregulates miR-29 expression, which reduces the expression of activation markers α-SMA, lysyl oxidase, and collagens (both type I and type III) in fibrotic mesenchymal cells and inhibits cell proliferation (Mannaerts et al., 2013). HDAC4 shows a complex distribution in various types of IPF bronchiolar cells, such as basal cells of hyperplastic (in the cytoplasm) or luminal ciliated bronchial cells (in the nucleus) (Korfei et al., 2015). These results indicate that HDAC4 is widely expressed in IPF fibroblasts and shows an array of intracellular signal transduction pathways, including but not limited to HDAC4/AKT/α-SMA, HDAC4/TGIF/α-SMA, and HDAC4/NOX4/α-SMA, depending on its subcellular localization.

#### 3.2.2 HDAC5

The human HDAC5 gene is located on chromosome 17q21, whereas the mouse HDAC5 gene exists on chromosome 11D11 (Verdel and Khochbin, 1999). HDAC5, with a molecular weight of 121.9 kDa, comprises 1122 amino acids and consists of C-terminal deacetylase and N-terminal adapter domains (Yang et al., 2021).

Currently, the role and function of HDAC5 in lung fibrosis are poorly understood. Korfei et al. showed that the expression of Class I and II HDACs, including HDAC5, is increased significantly in fibroblast foci and abnormal bronchiolar epithelium (Korfei et al., 2015). Aberrant down-expression of HDACs, especially HDAC2, HDAC5, and HDAC8, regulates the production of proinflammatory cytokines in alveolar macrophages in patients with chronic obstructive pulmonary disease (Ito et al., 2005). HDAC5 knockdown significantly inhibits the phosphorylation of Smad2/3 but upregulates Smad7 expression in TGF-β1-stimulated fibroblasts. This process relies on the interaction with myocyte enhancer 2A (Gao et al., 2022). Chromatin immunoprecipitation studies showed that nuclear factor erythroid 2-related factor 2 is an important antifibrotic gene and that its suppression state can be restored by HDAC inhibitor treatment in fibroblasts (Sanders et al., 2011). Increased HDAC5 expression in pathological cardiomyocyte hypertrophy decreases cardiomyocyte oxidative stress and represses NRF2 activation (Hu et al., 2019). Moreover, the downregulation of HDAC5 expression upregulates α-SMA expression in TGF-β1-induced IPF fibroblasts (Jones et al., 2019). Taken together, these studies showed that HDAC5 promotes the activation of fibroblasts and production of fibrotic factors; however, its role in IPF remains to be evaluated in experimental fibrosis.

#### 3.2.3 HDAC6

The human HDAC6 gene is located on chromosome Xp11, whereas the mouse HDAC6 gene exists on chromosome XA1. HDAC6 is the largest HDAC protein, with 1216 amino acids (Grozinger et al., 1999). It is a unique HDAC enzyme composed exclusively of two functional N-terminal catalytic domains and a C-terminal binding domain (Li et al., 2011).

HDAC6 is generally located in the cytoplasm but can shuttle into the nucleus. It is a cytoplasmic and cytoskeleton-associated HDAC that regulates the deacetylation of peroxiredoxin, β-catenin, and heat shock protein 90 (Parmigiani et al., 2008; Li et al., 2013; Liu et al., 2021). PRDX1 deficiency enhances EMT, lung fibroblast proliferation, and fibrosis progression through the PI3K/Akt and JNK/Smad signaling pathways (Sun et al., 2023). Heat shock protein 90 contributes to the TGF-β signaling activation-induced ECM synthesis causing fibrosis, increased inflammatory response, and lung impairment (Štorkánová et al., 2021; Roque, 2022). Remarkable HDAC6 upregulation and α-tubulin deacetylation occur in primary IPF fibroblasts (Korfei et al., 2015). HDAC6 expression is also significantly upregulated, along with a significant degree of α-tubulin deacetylation, in TGF-β1-stimulated fibroblasts (Saito et al., 2017). HDAC6 inhibitors (trichostatin A [TSA]), TSA is classified in Class 1/2 or pan HDAC inhibitor, are innovative and targeted disease-modifying agents in rare diseases, including IPF (Brindisi et al., 2020). Campiani et al. also reported that novel hHDAC6 inhibitors (6a-m) can effectively inhibit fibrotic sphere formation and cell viability, reversing the IPF phenotype (Campiani et al., 2021). In summary, these results suggest that HDAC6 overexpression in lung fibroblasts induces PF.

#### 3.2.4 HDAC7

The human HDAC7 gene is located on chromosome 12q13 and encodes a polypeptide of 912 amino acids, whereas the mouse HDAC7 gene is found on chromosome 15F1 and shares 95% similarity of amino acid sequence with human HDAC7 (Fischle et al., 1999; Fischle et al., 2001).

Recently, HDAC7 has been viewed as a key risk factor in enhancing the TGF-β-mediated transdifferentiation of fibroblasts into myofibroblasts during PF progression. Dakota et al. demonstrated that silencing HDAC7 significantly decreases the expression of profibrotic mediators NOX4 and CTGF but increases the expression of PGC1A in TGF-β-induced IPF fibroblasts (Jones et al., 2019). Additionally, HDAC7 knockdown can effectively inhibit SMAD signaling activation, fibroblast-myofibroblast transdifferentiation, and ECM production in TGF-β induced fibroblasts (Kang et al., 2018). HDAC7 could play a key role in airway fibrosis, which consists of p300 and AP-1 to form the transcriptional complex and increase CTGF expression in lung fibroblasts (Hua et al., 2021). Darren et al. reported that HDAC7 plays a vital role in cystic fibrosis by influencing the function of the cystic fibrosis transmembrane conductance regulator in human primary airway epithelia (Hutt et al., 2010). Silencing HDAC-7 significantly decreases the production of collagen-I, collagen-III, and profibrotic mediators ICAM-1 and CTGF in TGF-β-treated skin fibroblasts (Hemmatazad et al., 2009). Taken together, these findings suggest that HDAC7 plays a crucial role in the aberrant expression of profibrotic molecules during fibrogenesis.

#### 3.2.5 HDAC9

The human HDAC9 is located on human chromosome 7p21, whereas the mouse HDAC9 gene exists on chromosome 12A3 (Zhou et al., 2001). The HDAC9 protein is composed of 1069 amino acids and can be subjected to alternative splicing to form at least 29 variant isoforms (Mahlknecht et al., 2002; Brancolini et al., 2021).

HDAC9 is important in the human physiological system and is involved in the development of various diseases, such as cancer, diabetes, atherosclerosis, cardiovascular diseases, and liver fibrosis (Das et al., 2023). Zhang et al. demonstrated that HDAC9 expression increases in mouse and human fibrotic kidneys, where it deacetylates STAT1, while promoting G2/M phase arrest and activating fibroblasts. Macrophage accumulation, G2/M phase arrest, and ECM protein production play crucial roles in this process, and treatment with the high-affinity HDAC9 inhibitor TMP195 can effectively alleviate the above phenomena (Zhang et al., 2023). These studies show that HDAC9 is a powerful target for developing novel treatment methods for organ fibrosis. Currently, the functions of HDAC9 in PF remain unclear. HDAC9 is located in the cytoplasm but can shuttle to the nucleus, and its expression is significantly increased in the cytoplasm of lung myofibroblasts. Immunohistochemical analysis revealed that HDAC9 expression regulates vascular smooth muscle cells and is significantly upregulated in IPF (Korfei et al., 2015). Silencing HDAC9 remarkably increases the expression levels of ACTA2 in TGF-β-induced IPF fibroblasts (Jones et al., 2019). Moreover, HDAC9 and isoform HDAC-related proteins play important roles in the transdifferentiation of fibroblasts into myofibroblasts and cell apoptosis resistance in lung fibroblasts. These studies suggest that HDAC9 plays crucial roles in lung fibrosis; however, further research is warranted to specify these roles.

#### 3.2.6 HDAC10

The human HDAC10 gene is located on chromosome 22q13, whereas the mouse HDAC11 gene exists on chromosome 15E3 (de Ruijter et al., 2003). HDAC10 is composed of 20 exons, including the N-terminal catalytic domain and the C-terminal domain rich in leucine, and 669 amino acids (Cheng et al., 2021). It is widely expressed in human tissues and cultured mammalian cells, including the liver, kidney, pancreas, spleen, heart, and lungs (Tong et al., 2002).

The Class IIB member HDAC10 is pancellular. Zhang et al. demonstrated that HDAC10 plays an important role in asthma-induced eosinophil airway inflammation (Zhang et al., 2016). It is also a potential pathogenic gene for emphysema (Choi et al., 2009). Thus, HDAC10 may be a potential therapeutic target for treating respiratory system diseases. Immunohistochemical analysis has shown that HDAC10 is highly expressed in myofibroblasts within fibroblast foci in IPF (Korfei et al., 2015). Surprisingly, silencing HDAC10 with RNAi has shown no significant effect on the expression of profibrotic genes, such as α-SMA and collagen-I, in TGF-β1-stimulated fibroblasts (Saito et al., 2017). Furthermore, Tian et al. demonstrated that the upregulation of HDAC10 expression can significantly improve PF and lung function in silicosis by attenuating oxidative stress, inflammation, and fibrotic lesions via the ROS/NF-κB pathway (Tian et al., 2023). However, the effects of HDAC10 overexpression in IPF fibroblasts need further investigation.

In summary, the potential effects of significantly upregulated expression of all six Class II HDACs in fibroblasts remain poorly elucidated. Studies using Class II HDAC selective inhibitors in various experimental models of PF have contributed to further understanding of the functions of overexpressed Class II HDACs in fibrotic lung fibroblasts. Novel inhibitors targeting HDAC6 are promising drugs for treating IPF.

### 3.3 Class IV HDACs in PF

#### 3.3.1 HDAC11

Since its first discovery in 2002, HDAC11 is the smallest and unique member of the HDAC family (Yanginlar and Logie, 2018). The human HDAC11 gene is located on chromosome 3p25, whereas the mouse HDAC11 gene exists on chromosome 6D1 (de Ruijter et al., 2003). HDAC11 has 347 amino acids and similar sequences to the catalytic core regions of Class I and II HDAC proteins (Gao et al., 2002; Thangapandian et al., 2012). HDAC11 is expressed in several organs and tissues. It is mainly distributed in the heart, kidneys, smooth muscles, skeletal muscles, and testes (Chen et al., 2022). Immunoblot analyses revealed robust HDAC11 upregulation in IPF *versus* control fibroblasts (Korfei et al., 2015). However, reports on the mechanism by which HDAC11 influences PF development are rare. Notably, HDAC11 suppresses the transcription of Kruppel-like factor 15 by activator protein two to mitigate unilateral ureteral obstruction-induced renal fibrosis (Mao et al., 2020). Further, the upregulation of HDAC11 is involved in the mechanisms by which glucocorticoid relieves allergen-driven airway inflammation in mice (Zhang et al., 2016). Taken together, these studies suggest that little is known about the functions of HDAC11 in IPF and that further attention is needed in the future.

## 4 Potential regulatory network of HDACs involved in PF

IPF is a fatal lung disease of unknown etiology and is viewed as an epithelial-driven disease. Specifically, aging alveolar epithelial cells, which are dysfunctional and constantly subjected to microinjuries, experience regenerative defects due to these persistent insults. This triggers abnormal interactions between the damaged epithelium and mesenchymal cells, ultimately causing an imbalance in the levels of pro-fibrotic and antifibrotic mediators. This imbalance inhibits the natural repair mechanisms for chronic fibrosis, fostering an environment that encourages excessive proliferation and hyperactivity of fibroblasts and myofibroblasts, thus affecting the normal repair process of chronic fibrosis (Stella and Balestro, 2015; Spagnolo et al., 2021). The profound biological complexity of IPF lies in the diverse array of cell types and signaling pathways that are intricately intertwined with the disease’s pathogenesis. These mechanisms include, but are not limited to, the dysregulation of epithelial repair functions, imbalance in host defense mechanisms, accelerated cellular senescence, skewed immune responses (particularly the abnormal activation of macrophage subsets), fibrogenic reactions closely linked to abnormal kinase activation, transforming growth factor-β (TGF-β) and its downstream profibrotic signaling cascades, and reactivation of developmental pathways. These biological processes collectively contribute to the onset and progression of IPF.

**FIGURE 2:**
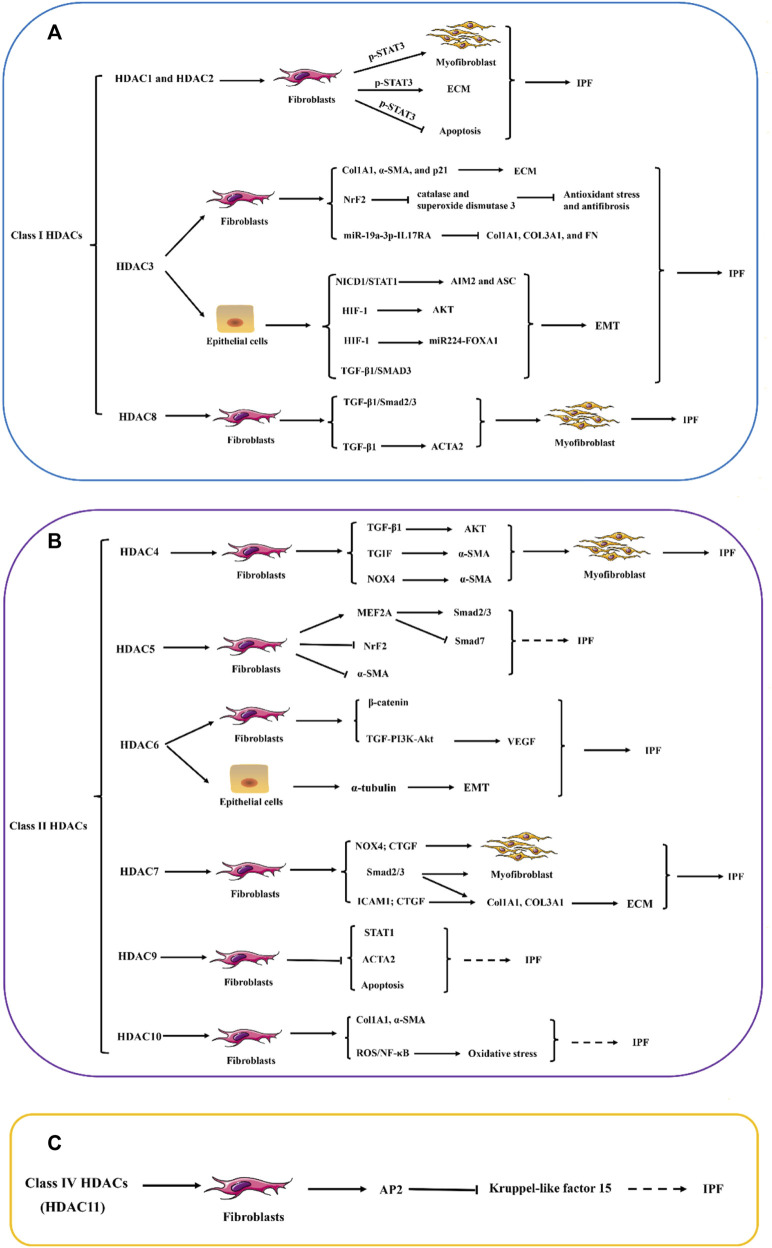
The potential role of HDAC enzymes in Idiopathic pulmonary fibrosis (IPF). **(A–C)** Abnormal increases in the expression of histone deacetylases (HDACs) in fibroblasts and myofibroblasts, as well as the aberrant expression of certain types of HDACs in epithelial cells, are believed to play a significant role in the pathogenesis of pulmonary fibrosis. These alterations in HDAC expression primarily influence the expression of numerous genes related to the ECM and anti-apoptotic pathways within cells. The dysregulation of HDACs affects the delicate balance of cellular processes, including EMT and FMD, which is a key feature of fibrotic lesions in the lungs. STAT1, signal transducer and activator of transcription 1; STAT3, signal transducer and activator of transcription 3; Col1A1, Collagen I; Col3A1, Collagen Ⅲ; α-SMA, α-smooth muscle actin; NrF2, Nuclear factor erythroid 2-related factor 2; IL17RA, Interleukin 17 Receptor A; NICD1, Notch intracellular domain 1; AIM2, Absent in melanoma 2; ASC, Apoptosis-related Dot-like Protein ASC; HIF-1, hypoxia inducible factor-1; FOXA1, Forkhead Box Protein A1; TGF-β1, Transforming growth factor beta 1; SMAD3, SMAD family member 3; ACTA2, actin alpha 2; TGIF, TGFB-induced factor; NOX4, NADPH oxidase 4; MEF2A, MADS box transcription enhancer factor 2; PI3K: Phosphatidylinositol 3-kinase; VEGF, vascular endothelial growth factor; CTGF, connective tissue growth factor; ICAM1, intercellular cell adhesion molecule-1; AP2, activator protein 2; NF-κB, nuclear factor kappa-B; ROS, reactive oxygen species; ECM, extracellular matrix; EMT, epithelial–mesenchymal transition FMD, Fibroblast-to-myofibroblast differentiation.

Within the realm of IPF, a critical function in fibrosis progression is played by the aberrant overexpression of Class I HDACs in fibroblasts and myofibroblasts. This abnormal expression is intricately linked to the activation of the TGF-β/SMAD2/3 signaling cascade and its downstream pathways, resulting in fibroblasts acquiring enhanced apoptosis resistance and transforming into myofibroblasts. During this transformation, a substantial amount of ECM, primarily represented by Col1A1 and α-SMA, is synthesized. Concurrently, the activation of the TGF-β1/SMAD3 and HIF-1 signaling pathways expedites the EMT of alveolar type 2 epithelial cells, thereby advancing the progression of lung fibrosis. Notably, in IPF fibroblasts, both the fibrotic-inducing activation of STAT3 and its lysine deacetylation exhibit a remarkable upregulation (Figure 2A). Compared to normal lung tissue, the myofibroblasts within the fibrotic lesions of IPF have significantly upregulated the protein levels of Class II HDACs. Under the stimulation of TGF-β, the lung fibroblasts enhance their a-SMA fiber formation and cell contractile ability, which is considered a crucial mechanism for promoting fibrosis. Class II HDACs play a significant role in the transdifferentiation process of fibroblasts into myofibroblasts and the apoptosis resistance of lung fibroblasts. This process involves the activation of multiple signaling pathways, including TGF-β/SMAD2/3, AKT/α-SMA, TGIF/α-SMA, NOX4/CTGF/α-SMA, and TGF-PI3K-Akt, which further promote the excessive production of ECM. Additionally, SMAD3 activation mediates the TGF-β-induced EMT, a process that is accompanied by the deacetylation of α-tubulin and the formation of mesenchymal stress fibers (Figure 2B). In contrast to Class I and Class II HDACs, our knowledge regarding the role and function of Class IV HDAC (HDAC11) in lung fibrosis is limited. Current research indicates that HDAC11 may be linked to the progression of lung fibrosis by suppressing the transcription of Kruppel-like factor 15 (KLF15) via the activation of protein 2 (Figure 2C).

## 5 HDAC inhibitors in IPF

**FIGURE 3:**
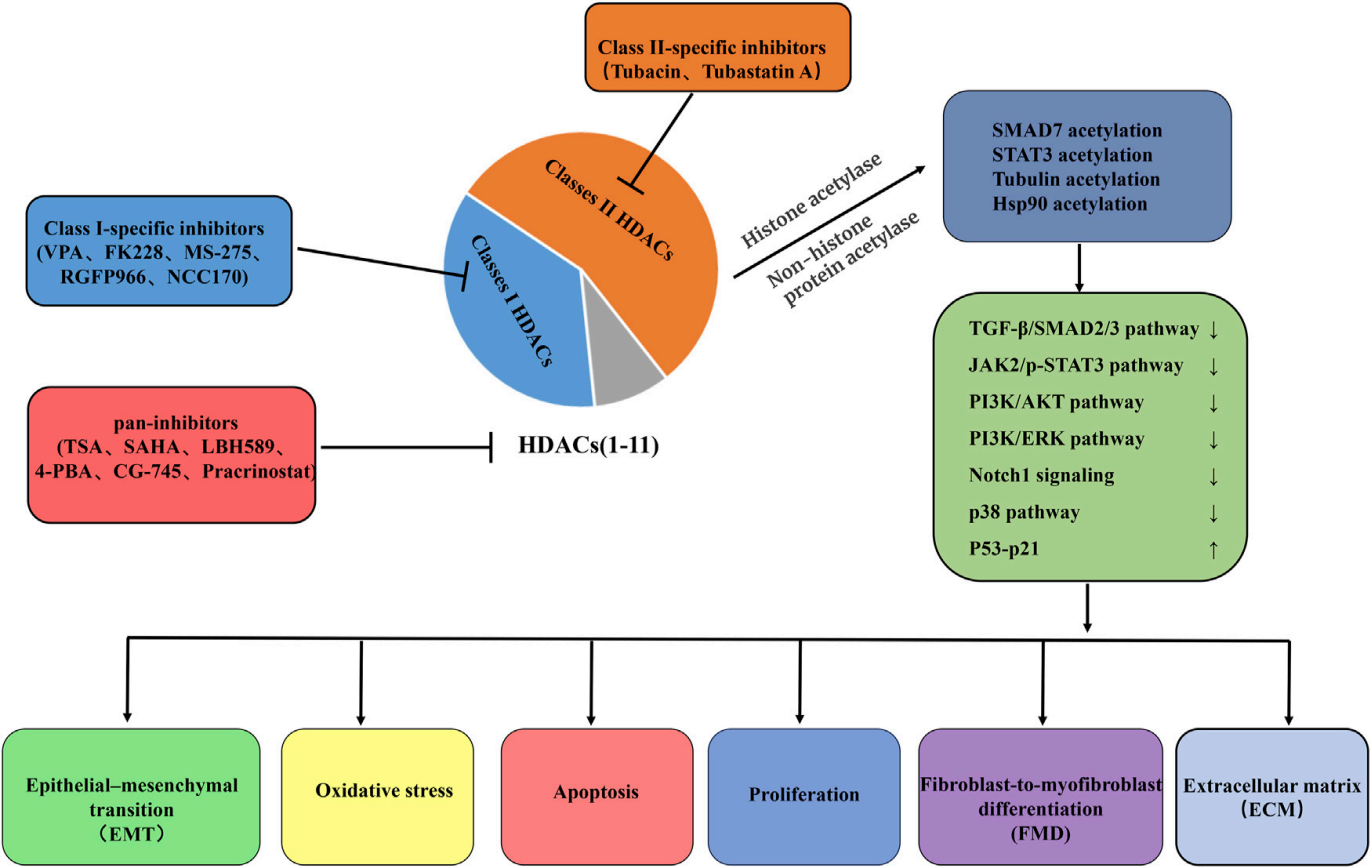
Summary of putative therapeutic effects of HDAC-inhibitor treatment on IPF. Pan-HDAC inhibition, Class I-specific inhibitors, and Class II-specific inhibitors influence the delicate balance of cell fate decisions, such as EMT, oxidative stress, cell proliferation, apoptosis, and FMD, processes that are critical in the remodeling of the ECM and the development of fibrosis. These beneficial effects were mediated largely through chromatin hyperacetylation and mechanisms involving non-histone protein acetylation. VPA, valproic acid; FK228, romidepsin; MS-275, entinostat; TSA, trichostatin A; SAHA, vorinostat; LBH589, panobinostat; 4-PBA, 4-phenyl-butyrate; TGF-β1, Transforming growth factor beta 1; SMAD3, SMAD family member 3; JAK2, Janus kinase 2; STAT3, signal transducer and activator of transcription 3; PI3K, Phosphatidylinositol 3-kinase; Notch1, Notch homolog 1; EMT, epithelial–mesenchymal transition; FMD, Fibroblast-to-myofibroblast differentiation; ECM, extracellular matrix.

Currently, several natural and synthetic compounds are known to exhibit inhibitory effects on HDACs. However, because HDAC inhibitors do not exhibit the same level of inhibition across all HDAC enzymes, these agents can be classified into pan-inhibitors, Class II-specific inhibitors, and Class I-specific inhibitors (Han et al., 2024). Moreover, due to their potent antitumor mechanisms, numerous pan-inhibitors and type I specific inhibitors have been hailed as successful anticancer drugs. These mechanisms primarily involve inducing apoptosis and autophagy (Gilardini Montani et al., 2017), thereby halting tumor cell cycles (Zhang et al., 2013), inhibiting angiogenesis (Mottamal et al., 2015), diminishing tumor cell mobility and migration, and ultimately enhancing the susceptibility of tumor cells to radiotherapy and chemotherapy (Lee et al., 2010). Since 2006, vorinostat (SAHA), the first-approved HDAC inhibitor for cancer treatment by FDA, has been utilized in clinical settings. Concurrently, significant research has been devoted to exploring various HDAC inhibitors in preclinical models of lung fibrosis/IPF. These inhibitors encompass: (a) hydroxamic acids such as TSA (a naturally occurring compound), SAHA, panobinostat (LBH589), and parcinostat (SB939); (b) cyclic peptides, notably romidepsin (FK228); (c) synthetic compounds such as 4-phenylbutyrate sodium, benzamide MS-275 (entinostat), and CG-745; and (d) subtype-specific HDAC inhibitors, including the short-chain fatty acid VPA, RGFP966, NCC170, tubacin, and tubastatin A. Table 1 encapsulates the overarching therapeutic impacts of diverse HDAC inhibitors on preclinical models pertaining to lung fibrosis/IPF. Overall, HDAC inhibitors either globally or selectively suppress the activity of HDACs, altering the acetylation levels of proteins such as SMAD7, STAT3, tubulin, and Hsp90 through both histone and non-histone acetyltransferases. This modulation regulates the activation of downstream signaling pathways, including TGF-β/SMAD2/3, JAK2/p-STAT3, PI3K/AKT, PI3K/ERK, Notch1, p38, and P53-p21. Subsequently, it affects processes like EMT, oxidative stress levels, apoptosis resistance in fibroblasts/myofibroblasts, the transdifferentiation of fibroblasts into myofibroblasts, and the excessive formation of ECM to varying degrees (Figure 3).

**TABLE 1:** HDAC inhibitors for treatment of pulmonary fibrosis.

HDAC inhibiton	Cells or tissue	Type of HDACs	Proposed function	Refs
trichostatin A (TSA)	lung fibroblasts of bleomycin mice, primary IPF fibroblasts, blood monocyte derived macrophages, TGF-β-treated human normal lung fibroblasts	pan-HDAC	Fas/FAS↓, Thy1 (CD90)↓, ACTA2↓, α-SMA ↓; EMT↓, HMGB1↑; FMD↓; alleviation of lung fibrosis	Glenisson et al. (2007), Guo et al. (2009), Sanders et al. (2011), Huang et al. (2013), Sanders et al. (2014), Ye et al. (2014), Ota et al. (2015), Zheng et al. (2024)
panobinostat (LBH589)	primary IPF fibroblast, TGF-β-treated IPF fibroblasts	pan-HDAC	COX2↓, PGC1A↓, CXCL10↓, STAT3↓; ECM↓; FMD↓	Coward et al. (2009), Coward et al. (2010), Korfei et al. (2015), Korfei et al. (2018)
vorinostat (SAHA)	primary IPF fibroblasts, TGF-β-treated normal human lung fibroblasts (HFL1), bleomycin mouse model	pan-HDAC	BAK↑, BID↑, BCL2L1↓, COL3A1↓; FMD ↓; SMAD7 acetylation and stabilization; SMAD3 dephosphorylation; alleviation of lung fibrosis s	Zhang et al. (2013a), Sanders et al. (2014), Rao et al. (2016)
4-phenyl-butyrate (4-PBA)	bleomycin mouse/rat model A549 cells, alveolar epithelial cells	Class I- and Class IIA-HDAC	IL6↓, TGF-β↓, TNF-α↓, α-SMA↓, Col1a1↓, Col1a2 ↓; oxidative stress ↓; EMT↓; alleviation of lung fibrosis	Zhao et al. (2015), Kabel et al. (2016), Delbrel et al. (2019), Gong et al. (2020), Qin et al. (2021)
CG-745	bleomycin mouse model, PHMG-induced lung fibrosis	Class I-HDAC + HDAC6	α-SMA↓, Col1a1↓; alleviation of lung fibrosis	Kim et al. (2019)
pracrinostat	TGF-β-treated primary IPF fibroblasts	pan-HDAC, except HDAC6	PGC1A↓, ACTA2↓, IL6↓, PDGFA↓; FMD↓	Jones et al. (2019)
valproic acid (VPA)	primary IPF fibroblasts, TGF-β-treated A549 cells, bleomycin mouse/rat model	HDAC1, HDAC2	BIRC5↓, Col1a1↓, SMAD2/3↓; cell proliferation ↓; oxidative stress ↓; EMT↓; alleviation of lung fibrosis	Korfei et al. (2015), Noguchi et al. (2015), Kabel et al. (2016), Chen et al. (2021b)
romidepsin (FK228)	TGF-β-treated primary IPF fibroblasts, bleomycin mouse model	HDAC1, HDAC2	ACTA2↓, COL3A1↓, LOX↓; FMD↓; ECM↓; alleviation of lung fibrosis	Conforti et al. (2017)
entinostat (MS-275)	TGF-β-treated embryonic mouse fibroblasts, TGF-β-treated normal human lung fibroblasts	HDAC1, HDAC3	Adam12↓, Timp1↓, SPARC↓; Inhibition of PI3K and ERK pathways	Barter et al. (2010), Kamio et al. (2017), Wang et al. (2020)
RGFP966	bleomycin mouse model	HDAC3	NrF2↑; E-cadherin↑; ECM↓; EMT↓	Chen et al. (2021a), Xiong et al. (2023)
NCC170	TGF-β-treated normal human lung fibroblasts, Bleomycin mouse model	HDAC8	PPARG↑, ECM↓; alleviation of lung fibrosis	Saito et al. (2019)
tubacin	TGF-β-stimulated A549 cells	HDAC6	E-cadherin↑, PAI1↓, COL1A1↓; Notch1 signaling↓, p38 pathway↓	Shan et al. (2008), Deskin et al. (2016)
tubastatin A	TGF-β-stimulated human normal lung fibroblasts, Bleomycin mouse model	HDAC6	PI3K-AKT pathway↓, FMD ↓ ECM ↓; alleviation of lung fibrosis	(Saito et al., 2017)
6a-m	TGF-β-stimulated human normal lung fibroblasts	HDAC6	α-SMA ↓, COL1A1↓, COL3A1↓	Campiani et al. (2021)
HDAC4 siRNA	TGF-β-treated human normal lung fibroblasts	HDAC4	ACTA2 ↓, α-SMA ↓ TGF-β signaling↓, FMD↓ AKT phosphorylation↓	Glenisson et al. (2007), Guo et al. (2009)
HDAC7 siRNA	TGF-β-treated primary IPF fibroblasts; TGF-β/endothelin-treated fibroblasts	HDAC7	ACTA2↓, CTGF↓, NOX4↓ PGC1A↓, AP-1↓, α-SMA ↓ FMD↓; ECM↓	Kang et al. (2018), Jones et al. (2019), Hua et al. (2021)

IPF: idiopathic pulmonary fibrosis; EMT: epithelial–mesenchymal transition; ECM: extracellular matrix; FMD: fibroblast-to-myofibroblast differentiation; siRNA: small interfering RNA; ↑: upregulation; ↓: downregulation.

In summary, the targeted inhibition of a specific HDAC has the potential to provide therapeutic advantages, whereas employing pan-HDAC inhibitors could elicit a more robust therapeutic response. Furthermore, HDAC inhibitors that are FDA-approved for cancer treatment may emerge as promising candidates for the treatment of IPF.

## 6 Conclusions and future perspectives

FDA-approved antifibrotic drugs, such as nintedanib and pirfenidone, can alleviate the progression of IPF to some extent. However, IPF cannot be completely cured, and novel therapeutic drugs or targets are needed. As a family of enzymes crucial for gene transcription and chromatin remodeling, HDACs have been considered a potential molecular target for treating PF. FDA-approved HDAC inhibitors have shown antifibrotic effects in BLM-induced PF animal models; however, their use in fibrotic diseases has not yet been approved (Lyu et al., 2019; Yoon et al., 2019). Additionally, the research and development of single HDAC selective inhibitors is still in its early stages. The effectiveness and selectivity of HDAC inhibitors need to be evaluated, and relevant lead compounds need to be modified to produce highly selective and powerful inhibitors. Despite their many limitations, HDACs remain attractive targets in developing strategies and drugs for treating PF owing to their therapeutic potential and effectiveness.
